# Cisplatin inhibits SIRT3-deacetylation MTHFD2 to disturb cellular redox balance in colorectal cancer cell

**DOI:** 10.1038/s41419-020-02825-y

**Published:** 2020-08-06

**Authors:** Xingyou Wan, Chao Wang, Zhenyu Huang, Dejian Zhou, Sheng Xiang, Qian Qi, Xinyuan Chen, Eyal Arbely, Chen-Ying Liu, Peng Du, Wei Yu

**Affiliations:** 1 State Key Laboratory of Genetic Engineering, School of Life Sciences, Zhongshan Hospital, Fudan University, Shanghai, 200438 China; 2 Department of Colorectal and Anal Surgery, Xinhua Hospital, Shanghai Jiao Tong University School of Medicine, Shanghai, 200092 China; 3 Shanghai Colorectal Cancer Research Center, Shanghai, 200092 China; 4 Department of Chemistry, Ben-Gurion University of the Negev, Beer-Sheva, 8410501 Israel; 5 The National Institute for Biotechnology in the Negev, Ben-Gurion University of the Negev, Beer-Sheva, 8410501 Israel

**Keywords:** Stress signalling, Acetylation

## Abstract

The folate-coupled metabolic enzyme MTHFD2 (the mitochondrial methylenetetrahydrofolate dehydrogenase/cyclohydrolase) confers redox homeostasis and drives cancer cell proliferation and migration. Here, we show that MTHFD2 is hyperacetylated and lysine 88 is the critical acetylated site. SIRT3, the major deacetylase in mitochondria, is responsible for MTHFD2 deacetylation. Interestingly, chemotherapeutic agent cisplatin inhibits expression of SIRT3 to induce acetylation of MTHFD2 in colorectal cancer cells. Cisplatin-induced acetylated K88 MTHFD2 is sufficient to inhibit its enzymatic activity and downregulate NADPH levels in colorectal cancer cells. Ac-K88-MTHFD2 is significantly decreased in human colorectal cancer samples and is inversely correlated with the upregulated expression of SIRT3. Our findings reveal an unknown regulation axis of cisplatin-SIRT3-MTHFD2 in redox homeostasis and suggest a potential therapeutic strategy for cancer treatments by targeting MTHFD2.

## Introduction

Folate metabolism, known as one-carbon (1C) metabolism, supplies a one-carbon group that is transferred to biomolecules, such as amino acids and nucleotides. These metabolites are required for nucleotide synthesis and methylation reactions^1^. Recent studies have shown that the mitochondrial one-carbon pathway is often reprogrammed in cancer cells^2^. MTHFD2 performs two main reactions in the one-carbon metabolism: the 5,10-methylene-THF (CH2-THF) dehydrogenase and 5,10-methenyl-THF (CH+-THF) cyclohydrolase. The generation of NADPH by MTHFD2 is able to overcome oxidative stress and maintain redox homeostasis, which are critical steps for tumor progression^3^. Enzymes of the one-carbon pathway, including MTHFD2, were markedly elevated in many cancers^4,5^. The one-carbon enzymes are activated by NRF2 or ATF4 through transcriptional regulation^6,7^. However, the upregulated mechanism of MTHFD2 by posttranslational modification remains unknown. Targeting MTHFD2 is a promising approach to design innovative therapeutic drugs for tumor treatment^8^.

Cisplatin (CAS No. 15663-27-1, MF-Cl2H6N2Pt; NCF-119875), or cis-diamminedichloroplatinum (II), is situated as a metallic (platinum) coordination compound with a square planar geometry. It is one of the most-effective chemotherapeutic agents that has been approved for the treatment of various malignant tumors^9^. The anticancer effects of cisplatin were explained by its capacity to crosslink with the purine bases on the DNA and form DNA inter-/intra-strand crosslinks in cancer cells, resulting in interfering with DNA repair mechanisms, causing DNA damage, and subsequently inducing apoptosis in cancer cells^10^. Oxidative stress is one of the most cytotoxic effects of cisplatin functions^11^.

Oxidative stress refers to the cellular effective antioxidant response owing to excessive reactive oxygen species (ROS) or oxidants^12^. Mitochondria contribute the maximum in the generation of ROS as it consumes ~80% of molecular oxygen during oxidative phosphorylation^13,14^. Hypoxia-inducible factor-dependent upregulation of the one-carbon metabolism enzyme SHMT2 and MTHFD2, promote mitochondrial serine catabolism and NADPH production to play the critically defensive mechanisms^15^. In this study, we identified that MTHFD2 is the novel deacetylation substrate of SIRT3. SIRT3 can directly interact with and deacetylates MTHFD2 at lysine 88 to influence the enzyme activity. More importantly, the deacetylation of MTHFD2 leads to increase enzymatic activity and upregulates cellular NADPH levels. Cisplatin is able to inhibit SIRT3 expression and increase acetylation of MTHFD2 which results in decrease NADPH levels and upregulate ROS levels. Our results uncover an unknown mechanism of cisplatin-induced mitochondrial sirtuins regulation of a one-carbon metabolic pathway and suggest a potential therapeutic strategy of antitumor chemotherapy through targeting MTHFD2 K88 acetylation.

## Results

### MTHFD2 is acetylated at K88

**Fig. 1:**
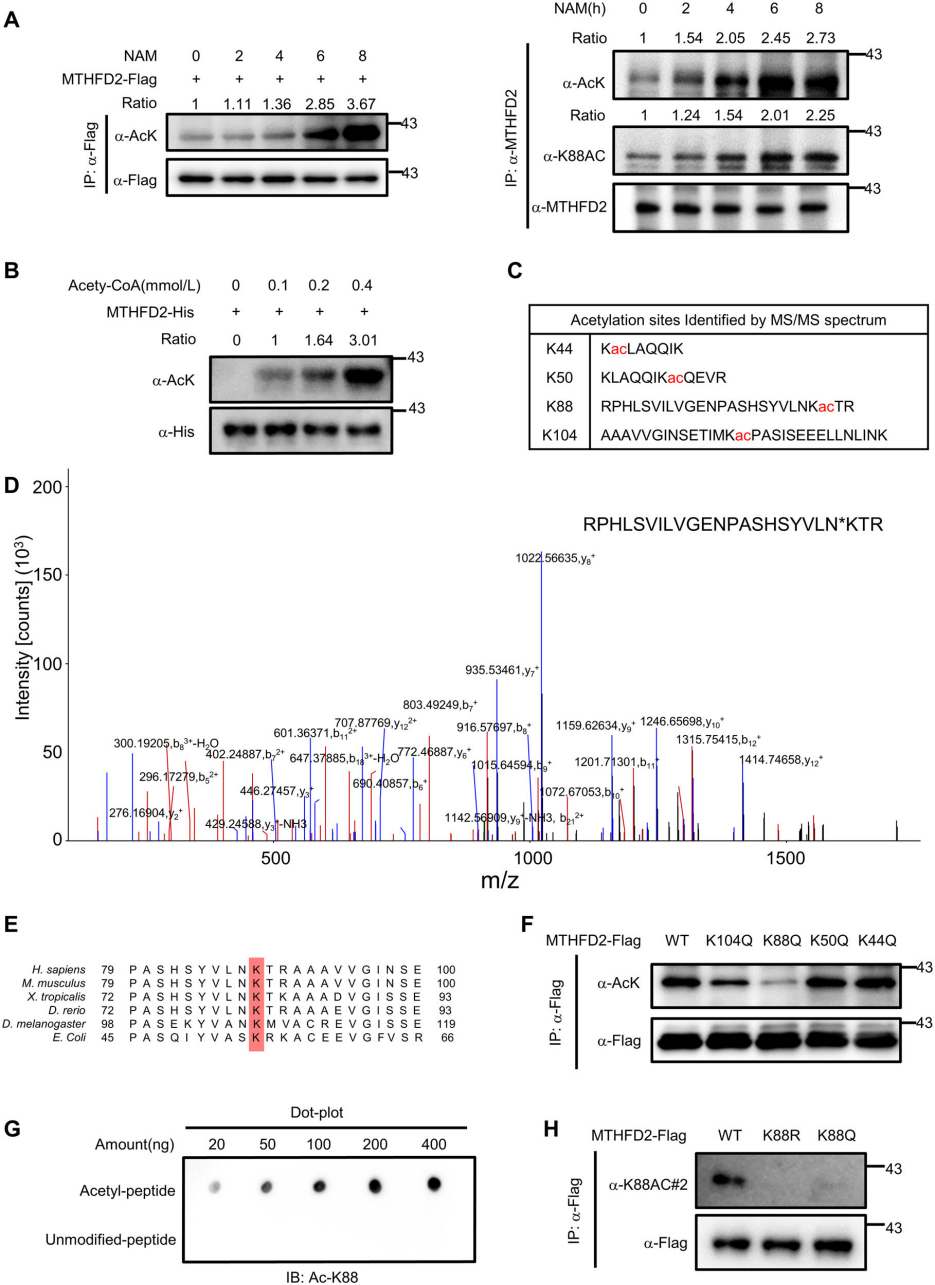
MTHFD2 is acetylated at K88. **a** Western blot detection of acetylation levels of ectopically expressed MTHFD2(left) and endogenous MTHFD2 (right) after treated with 5 μm NAM for the duration indicated. Flag-MTHFD2 was immunoprecipitated from cell lysate and its acetylation was examined with a pan-acetylated lysine antibody (α-AcK). Endogenous MTHFD2 acetylation was examined with pan-acetylated lysine antibody (α-AcK) and site-specific K88 acetylation antibody (Ac-K88). Relative MTHFD2 acetylation was normalized by Flag protein or endogenous MTHFD2. **b** In vitro MTHFD2 acetylation assay. MTHFD2 proteins were incubated with different concentrations of acetyl-CoA as indicated. Protein acetylation level was analyzed, and relative MTHFD2 acetylation was normalized by His protein. **c** Identification of acetylated MTHFD2 peptides by tandem mass spectrum. Identified sites were shown in chat. **d** Acetylated MTHFD2 K88 was identified by a tandem mass spectrum. The identified peptide is shown. **e** K88 in MTHFD2 is evolutionarily conserved. The sequences around MTHFD2 K88 from different species were aligned. **f** K88 is the major acetylation residue of MTHFD2. Flag-tagged WT MTHFD2 or mutants (K44Q, K50Q, K88Q, and K104Q) were expressed in HEK293T cells, followed by treatments with or without 5 μm NAM. Flag-MTHFD2 was immunoprecipitated and its acetylation was examined with α-AcK. **g** Characterization of anti-acetyl-MTHFD2 (K88) (α-acK88) antibody. Specificity of antibody against acetylated K88 residue of MTHFD2 was determined by dot blot assay. Nitrocellulose membrane was spotted with different amounts of acetyl-K88 peptide or unmodified peptide and immunoblotted with anti-acetyl-MTHFD2 (K88) antibody. **h** Characterization of acetyl-MTHFD2 (K88) antibody. Acetylation level of MTHFD2-Flag, MTHFD2-K88R-Flag, or MTHFD2 K88Q-Flag ectopically expressed in HCT116 cells was measured by the site-specific K88 acetylation antibody (Ac-K88).

The one-carbon enzymes, including MTHFD2, are activated by NRF2 or ATF4 through transcriptional regulation^6,7^. Although mass spectrometry-based proteomic analyses have identified several potentially acetylated sites of MTHFD2, whether the acetylation modification regulating the function of MTHFD2 remains unknown. To confirm that MTHFD2 is acetylated in cells, we first overexpressed full-length human MTHFD2 in HEK293T cells following treating with nicotinamide (NAM), a common deacetylase inhibitor against the Sirtuin family^16^. Flag-tagged MTHFD2 was immune precipitated and its acetylation level was tested by western blot with a pan-anti-acetylated lysine antibody (left). Endogenous MTHFD2 acetylation was examined with pan-acetylated lysine antibody (α-AcK) and site-specific K88 acetylation antibody (Ac-K88) (right). The result showed that MTHFD2 was acetylated and the acetylation was significantly enhanced under the treatment of nicotinamide in a dose-dependent manner (Fig. 1a). To confirm the MTHFD2 is acetylation in vitro, concentration increased acetyl-CoA is incubated with MTHFD2 in the in vitro acetylation reaction^17^. The acetylation levels of MTHFD2 were increased in a dose-dependent manner (Fig. 1b). To identify the major acetylated functional sites of MTHFD2, we purified the acetylated MTHFD2 from HEK293T cells and performed mass spectrometry assay. The mass spectrometric analysis suggested four putative acetylation lysine (K) residues in MTHFD2, including K44, K50, K88, and K104 (Fig. 1c, d). Sequence alignments in different species from *Escherichia coli* to *Homo sapiens* revealed that that K88 is evolutionarily conserved, indicating the potentially critical role for K88 in the function of MTHFD2 (Fig. 1e). Mutating these four lysines to arginine (to mimic deacetyl-modification) (K44R, K50R, K88R, K104R) and Glutamine (to mimic acetyl modification) (K44Q, K50Q, K88Q, K104Q)^18^ and then performed by western blot with an anti-pan-acetyllysine antibody. The results showed that the K88R/Q mutant exhibited significantly reduced overall acetylation levels of MTHFD2 (Fig. 1f and Supplemental Fig. 1a). To confirm K88 acetylation in vivo, we generated an antibody that specifically recognizes acetylated K88 in MTHFD2 through the K88-acetylated peptides of MTHFD2. Dot blot assay showed that the ac-K88 antibody preferentially recognized the K88-acetylated peptides but not the unacetylated control peptides (Fig. 1g), demonstrating the good specificity of this generated antibody. Using this site-specific antibody, a strong signal for ectopically expressed WT MTHFD2 was detected by western blot, but no signal for the K88R mutant was observed (Fig. 1h). These data show that MTHFD2 is acetylated both in cells and in vitro and lysine 88 is the major acetylation site of MTHFD2.

### SIRT3 is the major deacetylase for MTHFD2

**Fig. 2:**
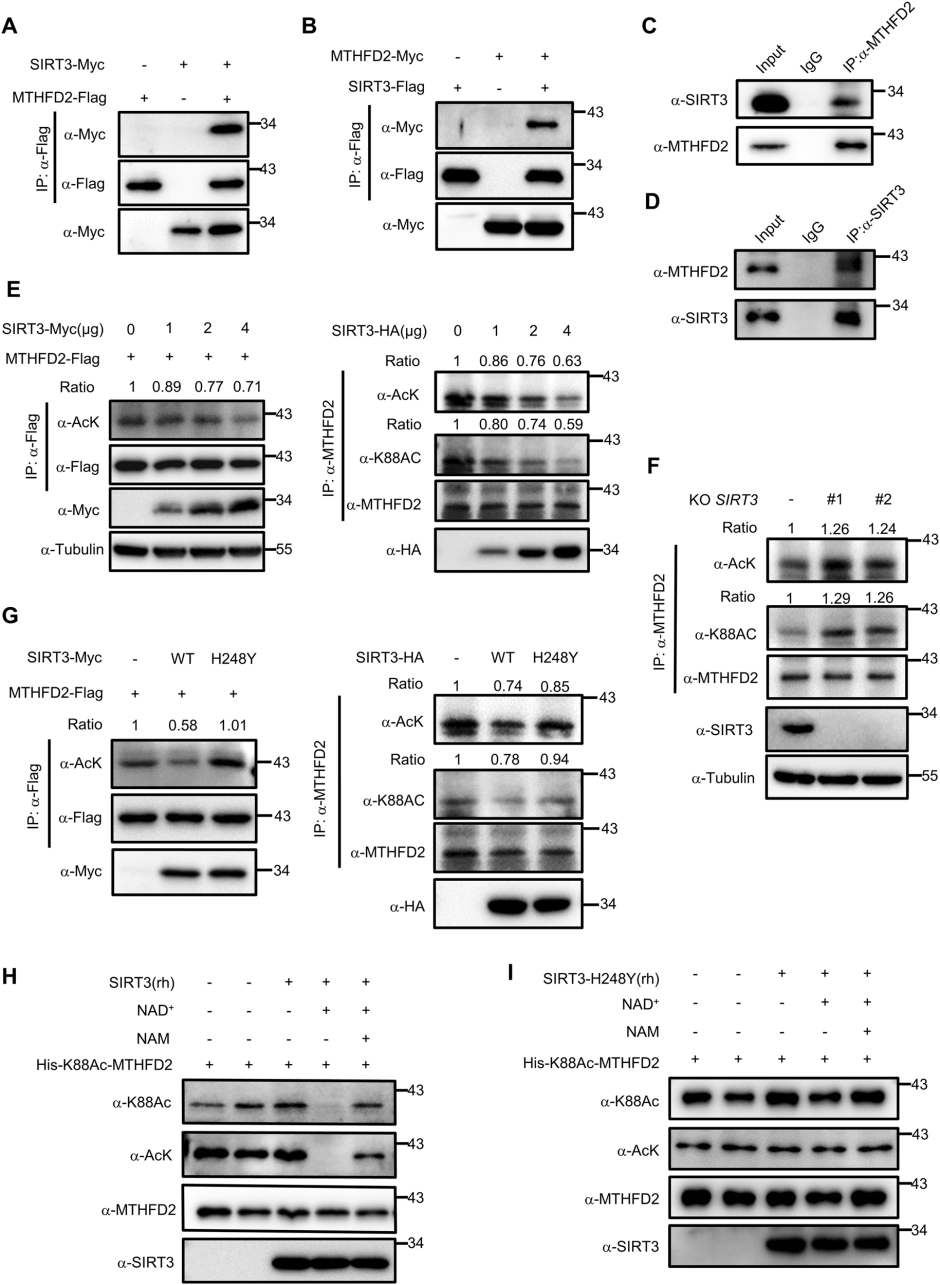
SIRT3 is the major deacetylase for MTHFD2. **a**, **b** SIRT3 interacts MTHFD2. Interactions between MTHFD2 and SIRT3 were determined when proteins were co-expressed with each other in HEK293T cells. **c**, **d** endogenous SIRT3 interacts with MTHFD2 in vivo. Whole-cell lysates were immunoprecipitated with control IgG, anti-SIRT3, or anti-MTHFD2 antibody, and the precipitated proteins were detected with anti-MTHFD2 or anti-SIRT3 antibody, respectively. **e** SIRT3 deacetylates MTHFD2 in a dose-dependent manner. Acetylation levels were detected of ectopically expressed MTHFD2(left) and endogenous MTHFD2 (right) after transfected with plasmids that express increasing amounts of SIRT3. Whole-cell lysates were immunoprecipitated with Flag-beads(left) or anti-MTHFD2 antibody(right), and the precipitated proteins were detected by an acetylation assay. **f** SIRT3-knockout increases MTHFD2 acetylation level. SIRT3-knockout generated by CRISPR/Cas9 were analyzed by western blotting for SIRT3 expression. Whole-cell lysates of HCT116 WT cells or SIRT3-knockout cells were immunoprecipitated with anti-MTHFD2 antibody, and the precipitated proteins were detected by an acetylation assay. **g** Acetylation levels were detected of ectopically expressed(left) and endogenous MTHFD2 (right) after transfected with empty vector or with SIRT3(WT) or SIRT3-H248Y (H248Y). Whole-cell lysates were immunoprecipitated with Flag-beads(left) or anti-MTHFD2 antibody(right), and the precipitated proteins were detected by an acetylation assay. **h** SIRT3 deacetylates MTHFD2 in vitro. Recombinant human (rh) SIRT3 deacetylates the site-specific K88-acetylated MTHFD2 proteins incubated with NAD+ or NAM, and acetylation level was determined by Ac-K88 and pan-AcK. **i** Recombinant catalytically inactive (rh) SIRT3 mutant H248Y and the site-specific K88-acetylated MTHFD2 incubated with NAD+ or NAM, and acetylation level was determined by Ac-K88 and pan-AcK.

As NAM treatment has been shown to increase MTHFD2 acetylation, implying that NAD^+^-dependent Sirtuins could be the deacetylase for MTHFD2. Mammalian SIRT1–3 display robust deacetylation activity, whereas SIRT4–7 have weak deacetylase activity and show activity toward other types of lysine modifications^19,20^. Given that both MTHFD2 and SIRT3 localizes in the mitochondria, we examined whether the major mitochondrial deacetylase SIRT3 could deacetylate MTHFD2 and affect its function^21^. Myc-tagged SIRT3 was co-expressed with MTHFD2-Flag in HEK293T cells, we found MTHFD2 interacted with SIRT3 (Fig. 2a). Reversible coIP confirmed that MTHFD2-Myc was pulled down by SIRT3-Flag (Fig. 2b). To examine the endogenous interaction of SIRT3 and MTHFD2, whole-cell lysates of HCT116 were incubated with anti-MTHFD2, anti-SIRT3 antibodies or control IgG. Then, the immune precipitated proteins were detected by anti-SIRT3 and anti-MTHFD2 antibodies. Endogenous SIRT3 was pulled down by endogenous MTHFD2 from cell lysates, but not from control IgG (Fig. 2c). Conversely, endogenous MTHFD2 was also co-precipitated with endogenous SIRT3 (Fig. 2d). We then explored the possibility that SIRT3 deacetylates MTHFD2 at the cellular level. SIRT3 deacetylated MTHFD2 in cells in a dose-dependent manner (Fig. 2e). To provide further insight into the role of SIRT3, we generated SIRT3-knockout cells using CRISPR/Cas9 technology and then endogenous MTHFD2 was immune precipitated in both SIRT3-WT HCT116 and SIRT3-KO HCT116 cells. The results showed that MTHFD2 acetylation levels were significantly increased in SIRT3-KO cells compared with SIRT3-WT cells (Fig. 2f). Acetylation levels were detected of ectopically expressed MTHFD2 (left) and endogenous MTHFD2 (right) after transfected with an empty vector or with SIRT3 (WT) or SIRT3-H248Y (H248Y). We found that the level of acetylated MTHFD2 was significantly low in overexpressed SIRT3 cells. Besides, SIRT3-H248Y, a SIRT3 enzymatically dead mutant^22^, failed to deacetylate MTHFD2 (Fig. 2g). Finally, we performed an in vitro deacetylation assay to determine whether SIRT3 directly deacetylates MTHFD2. A decrease in the level of K88Ac-MTHFD2 was only observed in the presence of both recombinant human SIRT3 and NAD^+^ as SIRT3 is an NAD^+^-dependent deacetylase. The deacetylation of K88Ac-MTHFD2 by SIRT3 was completely blocked by NAM treatment (Fig. 2h). In addition, the catalytically inactive recombinant human SIRT3-H248Y mutant could not deacetylate K88Ac-MTHFD2 despite the presence of NAD^+^ (Fig. 2i). Together, MTHFD2 is a novel target of SIRT3.

### Cisplatin inhibits SIRT3 expression to induce hyperacetylation of MTHFD2

**Fig. 3:**
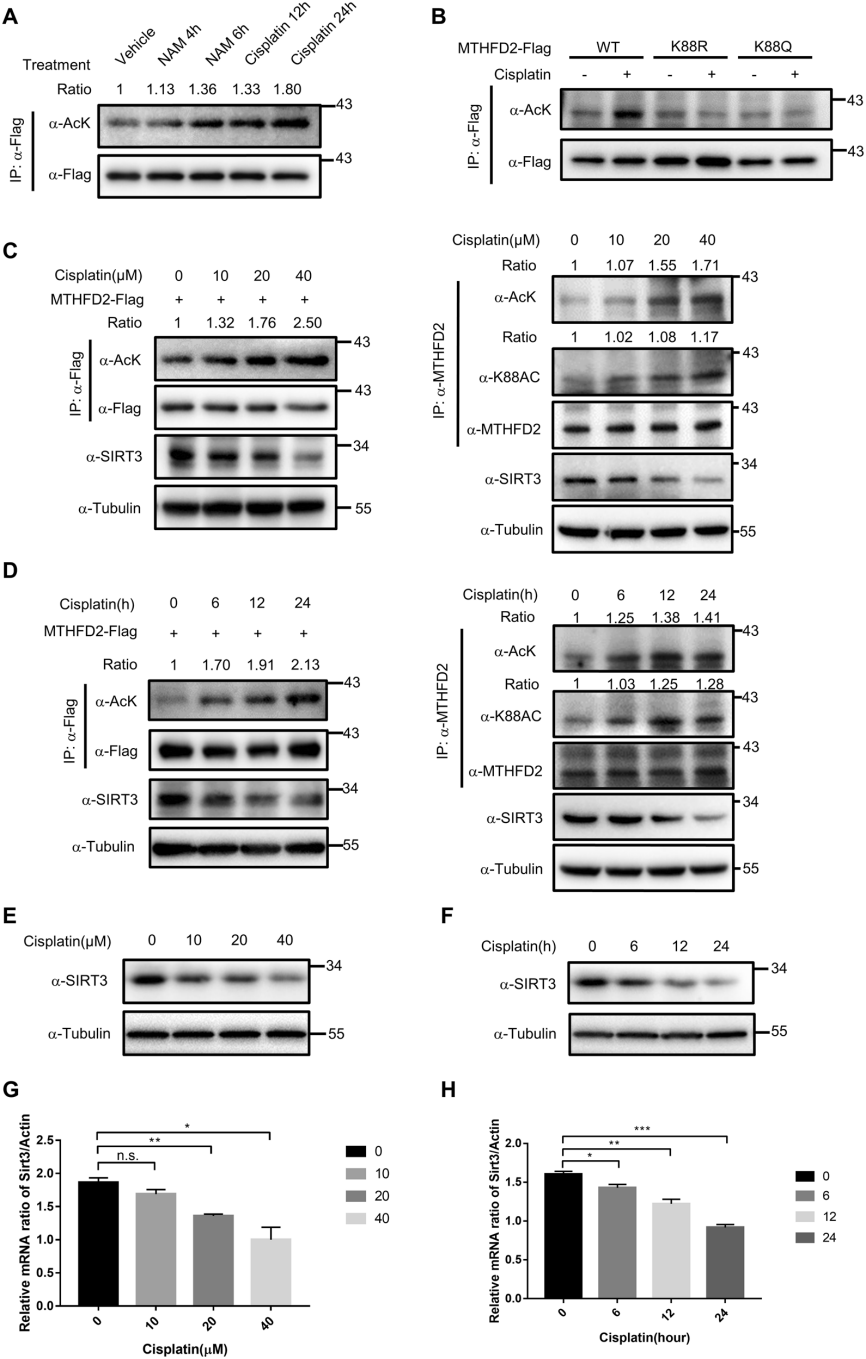
Cisplatin inhibits SIRT3 expression and induces acetylation of MTHFD2. **a** Cisplatin-induced MTHFD2 K88 acetylation. Flag-tagged MTHFD2 was expressed, which were then treated with NAM (5 μm) for 4 or 6 h and cisplatin (40 μm) for 12 or 24 h. Flag-MTHFD2 was immunoprecipitated with Flag beads and immunoblotting was performed with the antibodies indicated. Relative acetylation was normalized by Flag protein. **b** Cisplatin treatment increases MTHFD2 WT acetylation levels but not K88R/K88Q mutant acetylation levels. Flag-tagged WT MTHFD2 or mutants (K88R and K88Q) were expressed in HCT116 cells, followed by treatments with or without 40 μm NAM for 24 h. Flag-MTHFD2 was immunoprecipitated and its acetylation was examined with α-AcK. **c**, **d** Cisplatin treatment increases MTHFD2 acetylation in a dose and time-dependent manner. Acetylation levels were detected of ectopically expressed MTHFD2(left) and endogenous MTHFD2 (right) after treated with cisplatin for the duration indicated at different concentrations for 24 h **c** or at a concentration of 40 μm for different time **d**. **e**, **f** Western blot analysis of SIRT3 protein levels in control and cisplatin-treated HCT116 cells. Cisplatin treatment decreases SIRT3 protein levels in a dose and time-dependent manner. HCT116 cells were treated with cisplatin for the duration indicated at different concentrations for 24 h **e** or at a concentration of 40 μm for different time **f**. **g**, **h** Real-time PCR analysis of the expression of SIRT3 mRNA. Cisplatin treatment decreases SIRT3 mRNA levels in a dose and time-dependent manner. HCT116 Cells were treated with cisplatin for the duration indicated at different concentrations for 24 h **g** or at a concentration of 40 μm for different time **h**, followed by statistical analysis. *P < 0.05; **P < 0.01; ***P < 0.001; ****P < 0.0001; n.s. not significant.

Cisplatin is currently one of the most-effective chemotherapeutic drugs used for treating various malignant tumors^23,24^. Besides the cytotoxic effect of cisplatin occurs via DNA damage and mitochondrial injury^10,24^, oxidative stress and impaired energy metabolism are likely associated with cisplatin^25^. SIRT3 is a key regulator of cell survival and defense in response to oxidative stress induced by various injuries^26^. Given our observation that SIRT3 deacetylate MTHFD2, we speculated that cisplatin might regulate SIRT3 to affect the acetylation of MTHFD2. To test this possibility, HEK293T cells were treated with cisplatin (40 μm) for 12 and 24 h. Interestingly, cisplatin treatment significantly induced MTHFD2 acetylation, similar with the treatment of 5 mm NAM (Fig. 3a). With the treatment of cisplatin, we found that the acetylation of wildtype MTHFD2 was significantly upregulated, whereas the K88R and K88Q mutants of MTHFD2 did not change the acetylation levels (Fig. 3b), implying that K88 was the critical site in response to cisplatin. We also found that the treatment of cisplatin increased MTHFD2 acetylation and K88Ac in a dose- and time-dependent manner (Fig. 3c, d). Interestingly, the protein levels of SIRT3 were significantly decreased (Fig. 3c, d). Moreover, we assayed whether cisplatin could decrease the protein expression of SIRT3 in a dose- or time-dependent manner. We found the protein of SIRT3 was markedly downregulated under cisplatin exposure in a dose- and time-dependent manner (Fig. 3e, f). Consistently, the mRNA expression of SIRT3 was significantly reduced in the cisplatin-treated HCT116 cells compared with control cells (Fig. 3g, h). Taking together, these results suggest that cisplatin induces MTHFD2 acetylation by downregulating SIRT3 expression and suppressing the SIRT3-deacetylation process.

### K88 acetylation inhibits MTHFD2 enzymatic activity

**Fig. 4:**
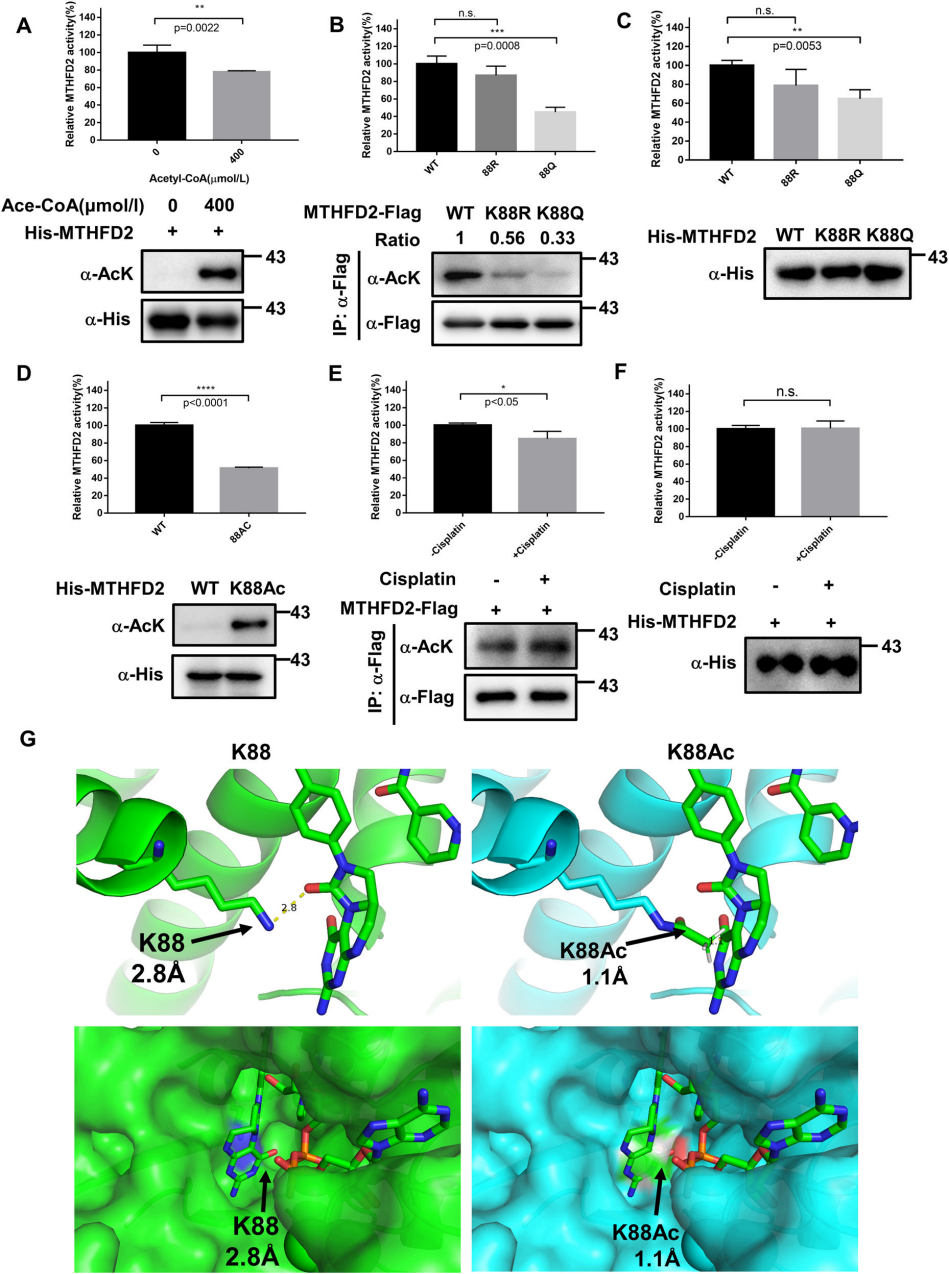
K88 acetylation reduces MTHFD2 enzymatic activity. **a** In vitro acetylation of MTHFD2 decreases its activity. MTHFD2 proteins were purified and incubated with or without 400 μm acetyl-CoA for 15 min at 30 °C before specific enzymatic assay. Error bars, ± SD (*n* = 3). *P < 0.05; **P < 0.01; ***P < 0.001; ****P < 0.0001; n.s., not significant for the indicated comparison. **b**, **c** K88Q mutation decreases MTHFD2 activity. MTHFD2 proteins were purified by immunoprecipitation from HCT116 cells **b** or recombinant expressed in *E. coli* and purified by nickel affinity chromatography **c**, and the enzyme activity assays were performed. Error bars, ± SD (*n* = 3). *P < 0.05; **P < 0.01; ***P < 0.001; ****P < 0.0001; n.s., not significant for the indicated comparison. **d** The site-specific K88-acetylated MTHFD2 decreases its enzyme activity. MTHFD2 and K88Ac-MTHFD2 were recombinant expressed and purified by nickel affinity chromatography and performed activity assay. Error bars, ± SD (*n* = 3). *P < 0.05; **P < 0.01; ***P < 0.001; ****P < 0.0001; n.s., not significant for the indicated comparison. **e** Cisplatin represses MTHFD2 activity by acetylation. HCT116 cells were transfected with Flag-tagged MTHFD2 and treated with or without Cisplatin for 24 h. Immunoprecipitated MTHFD2 activity was detected by specific enzymatic assay. Error bars, ± SD (*n* = 3). *P < 0.05; **P < 0.01; ***P < 0.001; ****P < 0.0001; n.s., not significant for the indicated comparison. **f** Cisplatin cannot repress MTHFD2 activity in vitro. MTHFD2 proteins were purified and incubated with or without 40 μm cisplatin for 15 min at 30 °C before specific enzymatic assay. Error bars, ± SD (*n* = 3). *P < 0.05; **P < 0.01; ***P < 0.001; ****P < 0.0001; n.s., not significant for the indicated comparison. **g** Molecular modeling of acetylation of K88 in MTHFD2 (left, WT; right, K88Ac) with K88 (PDB ID 5TC4) bounded with LY345899 (an inhibitor of MTHFD2 similar to substrate). The WT MTHFD2 is shown in green chains, K88Ac is shown in blue chains and LY345899 is shown in stick format. Bottom view is enlarged to show that LY345899 binds to K88 of MTHFD2.

To investigate the physiologic functions of the acetylation regulation of MTHFD2, we first examined its effect on MTHFD2 enzymatic activities. We performed the In vitro acetylation assay of recombination MTHFD2 and the data showed that hyperacetylated MTHFD2 significantly decreased its enzymatic activity (Fig. 4a). To confirm whether K88 acetylation negatively regulates the enzymatic activity of MTHFD2, WT, K88R, and K88Q mutant of MTHFD2 were overexpressed in HCT116 cells and *E. coli*. Then the enzymatic activity was measured by using optimized methods. We found that K88Q, acetylation-mimic mutant, showed ~70% activity of MTHFD2 (Fig. 4b) but K88R-deacetylation mimic mutant retains substantial enzymatic activity, indicating that K88 acetylation inhibits MTHFD2 activity. WT, K88R, and K88Q mutants of MTHFD2 from *E. coli* showed similar results from HCT116 (Fig. 4c). These results indicate that the positive charge of Lys 88 is critical for MTHFD2 catalytic activity. Although the mutation of lysine to arginine or glutamine can provide general insight into possible functional effects of protein acetylation, such strategies do not reveal the true consequences of acetylation^27^. To definitively demonstrate the effect of K88 acetylation on MTHFD2 activity, we utilized site-specific incorporation assay to prepare recombinant Nε-acetyl-lysine at position 88 of MTHFD2 in *E. coli*^28^. The incorporation of Nε-acetyl-lysine was genetically encoded through an amber stop codon (TAG) at amino acid position 88. The mutant version of MTHFD2 was co-expressed in *E. coli* with an orthogonal Nε-acetyl-lysine-tRNA syntheses. Only with Nε-acetyl-lysine in the growth medium was the full-length protein formed. This expression system produced MTHFD2 proteins with 100% acetylation at K88. Like the acetyl-mimetic mutation of Lys to Gln, the MTHFD2-K88Ac exhibited low enzymatic activity (Fig. 4d). In addition, cells treated with cisplatin, which increased MTHFD2 acetylation levels and reduced the MTHFD2 activity (Fig. 4e). However, in vitro incubation of cisplatin with MTHFD2 cannot repress the activity of MTHFD2 (Fig. 4f), indicating that cisplatin might indirectly affect the acetylation and activity of MTHFD2. Collectively, these results demonstrate that acetylation at lysine 88 inhibits MTHFD2 activity under cisplatin treatment in cells.

We attempted to provide the structural evidence of WT MTHFD2 and MTHFD2-K88Ac to reveal the potential molecular mechanism. Luckily, Robert Gustafsson et al.^8^ resolved the crystal structure of human MTHFD2 in 2016. The crystal structure of MTHFD2 showed that K88 resides near the bound substrate of tetrahydrofolate (THF), whereas the molecular docking analysis showed that addition of acetyl group in the MTHFD2 K88 is able to clash with the THF in space (Fig. 4g). This may illustrate the potential mechanism of how acetylation at K88 in MTHFD2 can regulate its enzymatic activity.

### MTHFD2 acetylation restrains cellular redox balance

**Fig. 5:**
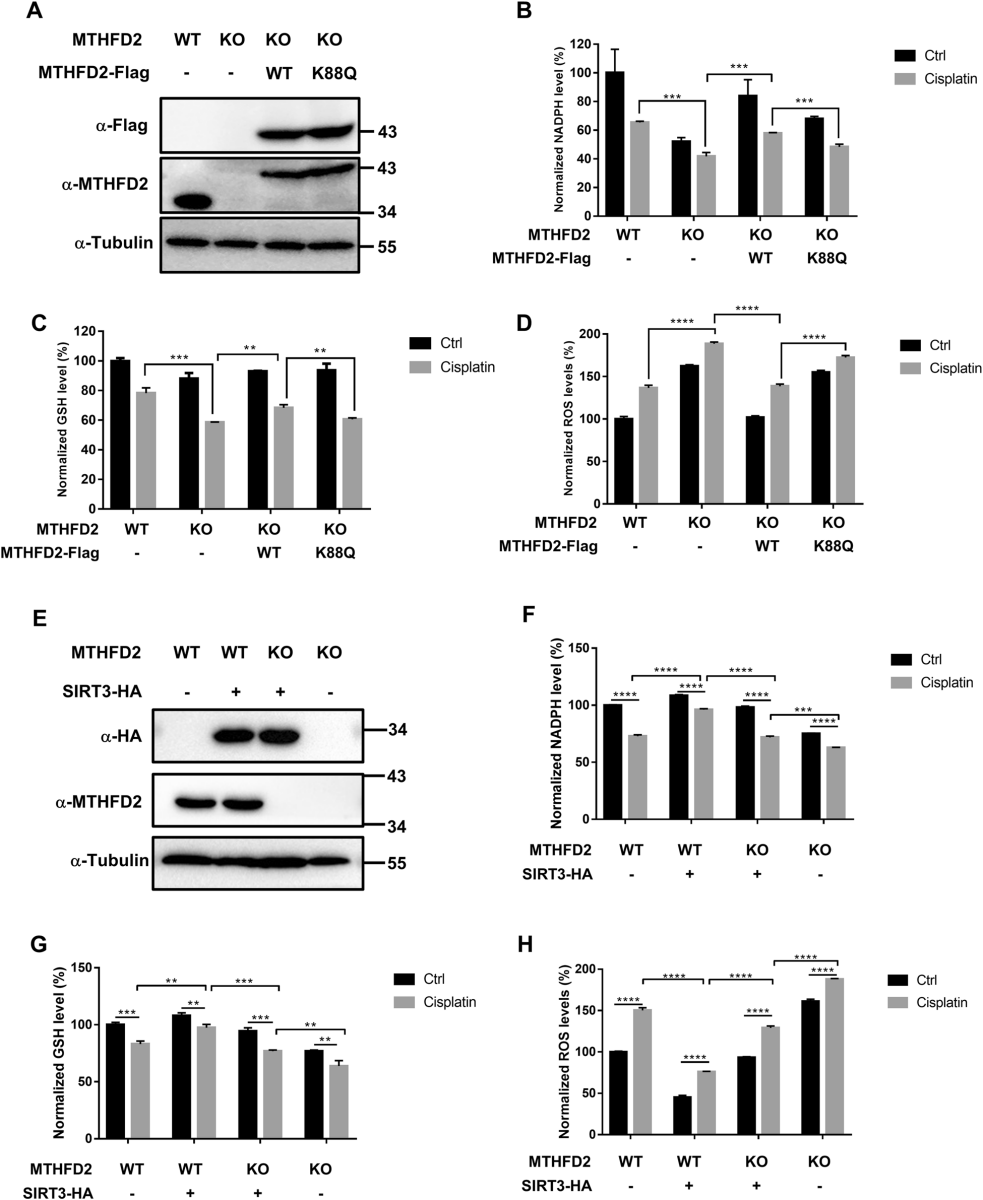
Acetylation of MTHFD2 at K88 restrains cellular redox balance. **a** Identification of HCT116 MTHFD2 rescued cell lines. MTHFD2 was knocked out in HCT116 cells, empty vector, Flag-tagged WT, and 88 K to Q mutant of MTHFD2 was stably re-expressed in MTHFD2 KO cells. Total lysates were prepared from four cell lines and detected by western blot analysis. **b** MTHFD2 K88Q decreases cellular NADPH level under cisplatin treatment. HCT116 WT cells, HCT116 KO cells, MTHFD2 WT cells, or MTHFD2 K88Q cells were treated in the presence or absence of cisplatin for 24 h. Cell’s cellular NADPH level was measured. Error bars, ± SD (*n* = 3). *P < 0.05; **P < 0.01; ***P < 0.001; ****P < 0.0001; n.s., not significant for the indicated comparison. **c** MTHFD2 K88Q decreases cellular GSH level under cisplatin treatment. HCT116 WT cells, HCT116 KO cells, MTHFD2 WT cells, or MTHFD2 K88Q cells were treated in the presence or absence of cisplatin for 24 h. GSH level in cells was measured. Error bars, ± SD (*n* = 3). *P < 0.05; **P < 0.01; ***P < 0.001; ****P < 0.0001; n.s., not significant for the indicated comparison. **d** MTHFD2 K88Q increases cellular ROS level under cisplatin treatment. HCT116 WT cells, HCT116 KO cells, MTHFD2 WT cells, or MTHFD2 K88Q cells were treated in the presence or absence of cisplatin for 24 h and ROS level was measured. Error bars, ± SD (*n* = 3). *P < 0.05; **P < 0.01; ***P < 0.001; ****P < 0.0001; n.s., not significant for the indicated comparison. **e** Identification of SIRT3 overexpression cell lines. Empty vector, HA-tagged SIRT3 was stably re-expressed in HCT116 cells and MTHFD2 KO cells. Total lysates were prepared from four cell lines and detected by western blot analysis. **f** SIRT3 rescues cellular NADPH level under cisplatin treatment. HCT116 cells, SIRT3 overexpression cells, SIRT3 overexpression-MTHFD2 KO cells, or MTHFD2 KO cells were treated in the presence or absence of cisplatin for 24 h. Cell’s cellular NADPH level was measured. Error bars, ± SD (*n* = 3). *P < 0.05; **P < 0.01; ***P < 0.001; ****P < 0.0001; n.s., not significant for the indicated comparison. **g** SIRT3 rescues cellular GSH level under cisplatin treatment. HCT116 cells, SIRT3 overexpression cells, SIRT3 overexpression-MTHFD2 KO cells, or MTHFD2 KO cells were treated in the presence or absence of cisplatin for 24 h. GSH level in cells was measured. Error bars, ± SD (*n* = 3). *P < 0.05; **P < 0.01; ***P < 0.001; ****P < 0.0001; n.s., not significant for the indicated comparison. **h** SIRT3 rescues the higher cellular ROS under cisplatin treatment. HCT116 cells, SIRT3 overexpression cells, SIRT3 overexpression-MTHFD2 KO cells, or MTHFD2 KO cells were treated in the presence or absence of cisplatin for 24 h and ROS level was measured. Error bars, ± SD (*n* = 3). *P < 0.05; **P < 0.01; ***P < 0.001; ****P < 0.0001; n.s., not significant for the indicated comparison.

To further examine the function of MTHFD2 K88 acetylation, we first generated MTHFD2-knockout HCT116 cells by the CRISPR/Cas9 technique. Several KO cell lines were obtained and confirmed (Supplemental Fig. 1b). The MTHFD2 KO#2 clone was chosen for further experiments. Using this clone, we generated three stably re-expressed cell lines: empty vector, WT, and K88Q mutant of MTHFD2. Both re-expressed WT and K88Q mutant of MTHFD2 had similar expression levels in HCT116 cells (Fig. 5a). NADPH is the substrate of MTHFD2, which has a critical role in cellular redox balance (Supplemental Fig. 1c). To evaluate the effect of MTHFD2 acetylation on cellular redox homeostasis, we performed the cellular redox analysis to measure the levels of NADPH, GSH, and ROS under cisplatin treatment. We found that MTHFD2 KO significantly reduced the NADPH levels and MTHFD2 WT could abrogate this reduction of NADPH. However, MTHFD2 K88Q failed to rescue the reduction of NADPH under cisplatin treatment (Fig. 5b). Similarly, when treated with cisplatin MTHFD2 KO reduced the GSH levels and MTHFD2 WT rescued the decreased GSH, but not in MTHFD2 K88Q (Fig. 5c). Consistently, MTHFD2 KO cells and MTHFD2 K88Q showed higher cellular ROS levels under cisplatin treatment than MTHFD2 WT (Fig. 5d). Furthermore, we identified the role of SIRT3 in this signal axis. Empty vector and HA-tagged SIRT3 was stably re-expressed in HCT116 cells and MTHFD2 KO cells (Fig. 5e). We performed the cellular redox analysis through measuring the levels of NADPH, GSH, and ROS under cisplatin treatment. We found that overexpression of SIRT3 significantly upregulated the reduction of NADPH and GSH levels and rescued the higher cellular ROS under cisplatin treatment (Fig. 5f–h). Moreover, SIRT3 still plays an antioxidative role in MTHFD2 KO cells compared without SIRT3 (Fig. 5f–h), because several antioxidative enzymes have been identified as the substrates of SIRT3, such as IDH2 and SOD2^12,14,29,30^. MTHFD2 KO in SIRT3 overexpression cells reduced the NADPH and GSH levels and showed higher cellular ROS levels under cisplatin treatment (Fig. 5f–h). Together, these results indicate that the SIRT3-MTHFD2 axis plays a critical role in cisplatin treatments.

### K88 acetylation of MTHFD2 is downregulated in CRC with high SIRT3 expression

**Fig. 6:**
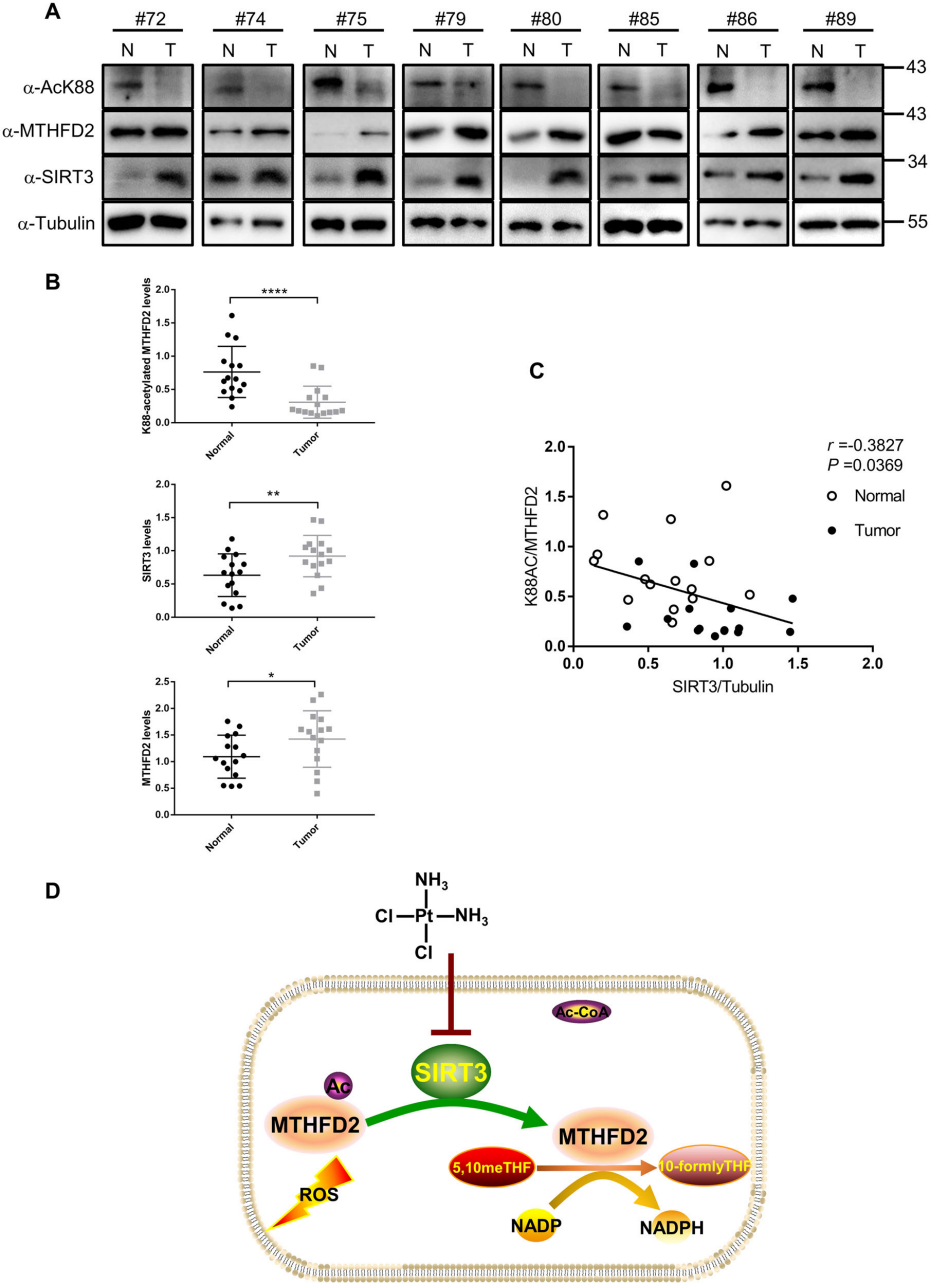
K88 acetylation of MTHFD2 is downregulated in CRC. **a** In total, 15 pairs of tumor tissues (T) and adjacent normal tissues (N) were lysed. Protein levels of MTHFD2-K88Ac, MTHFD2, and SIRT3 were determined by direct western blot. Relative protein levels were normalized by α-Tubulin. Shown are eight pairs of samples. **b** Quantification of relative MTHFD2, MTHFD2-K88Ac and SIRT3 protein levels in the 15 pairs of samples tested. The intensities of indicated proteins were quantified using the ImageJ software, followed by statistical analysis. *P < 0.05; **P < 0.01; ***P < 0.001; ****P < 0.0001; n.s., not significant for the indicated comparison. **c** SIRT3 protein levels show negative correlation with MTHFD2-K88Ac. Correlation between MTHFD2 protein levels and MTHFD2-K88Ac levels in the tested 15 pairs of samples. Statistical analyses were performed with *F* test. **d** Working model. Acetylation at K88 under cisplatin treatment decreases SIRT3 expression, increases MTHFD2 acetylation level and inhibits MTHFD2 enzyme activity. In CRC, high SIRT3 levels promotes deacetylation of MTHFD2 to maintain its high activity and restraining cellular redox balance.

Our findings demonstrate that acetyllysine 88 impaired the enzyme activity of MTHFD2 to restraining cellular redox balance in human colorectal tumor cells. These results prompted us to investigate the protein expression levels of K88 acetylation in MTHFD2, MTHFD2, and SIRT3 in human colorectal tumors. We performed a western blotting analysis in the 15 pairs of colorectal tumor samples (T) and adjacent nontumor tissues (N) (Fig. 6a and Supplemental Fig. 1d). Of these 15 pairs of samples, MTHFD2 protein was significantly increased in tumor samples, however the acetylated K88 in MTHFD2 was reversibly downregulated in all tumor samples (*P* < 0.0001) (Fig. 6b). Most tumor samples with highly expressed MTHFD2 exhibited the upregulated protein levels of SIRT3 compared with adjacent nontumor tissues (*P* < 0.01) (Fig. 6b). Moreover, SIRT3 protein levels were significantly negatively associated with acetylated K88 MTHFD2 in our tissue pairs (*r* = −0.3827, *P* = 0.0369, Fig. 6c). Together, these results indicate that K88 acetylation of MTHFD2 is downregulated in colorectal tumors and is correlated with highly expressed SIRT3. Thus, K88 acetylation of MTHFD2, MTHFD2 protein and SIRT3 may be potential screening biomarkers for human colorectal tumors.

## Discussion

SIRT3 acts as the major NAD^+^-dependent deacetylase in mitochondria has attracted more attention recently. Studies reveal that SIRT3 deacetylates several enzymes to control their cellular metabolism pathways, including detoxification, β-oxidation, TCA cycle, and oxidative phosphorylation^29,31–33^. As a NAD(P)-dependent enzyme, MTHFD2 has an essential role in folate-mediated one-carbon metabolism in mitochondria^34^. This study provides an interesting example of insight into how acetylation regulates MTHFD2 enzyme activity. Here, we identified MTHFD2 as a novel target of SIRT3 and lys88 was the major acetylation site of MTHFD2. Our findings provide another piece of the puzzle in the holistic picture of cisplatin inducing tumor cell death by inhibiting SIRT3 regulated one-carbon metabolism (Fig. 6d). Understanding how MTHFD2 is involved in cellular redox balance is important to effectively develop therapeutics targeting this enzyme for cancer treatment.

Recent studies have revealed that the mitochondrial one-carbon pathway is essential for maintaining NADPH/NADP^+^ redox homeostasis^34,35^. MTHFD2 directly generates NADPH from NADP^+^ in this pathway, suggesting it could play a critical function of detoxification. In this study, we elucidated the mechanism of post-translational modifications in the regulation of MTHFD2 in cisplatin disturbing cellular redox balance. Our molecular structural mimic revealed that acetylation at K88 inhibited MTHFD2 enzymatic activity through decreasing its affinity toward the substrate 5,10-meTHF. We also showed that acetylated MTHFD2 leads to the inhibition of NADPH production, the upregulation of the cellular ROS, which might play a vital role in the cell growth of colorectal cancer.

Cisplatin is one of the most-effective chemotherapeutic agents in treating of various malignant tumors^9,11^. Mitochondria have been shown to be the primary target for cisplatin-induced oxidative stress, resulting in loss of sulfhydryl group in mitochondrial protein, inhibition of calcium uptake and reduction of mitochondrial membrane potential^36–38^. SIRT3 is the major deacetylase in mammalian mitochondria^39^. SIRT3 has been shown to deacetylate and increase the activities of antioxidant enzymes and enhance the clearance of mitochondrial ROS, thereby maintaining cellular redox homeostasis^26,40^. In our study, we revealed that MTHFD2 is a novel substrates of SIRT3, which results in keep MTHFD2 enzyme activity and NADPH production in tumor cells. We found that cisplatin inhibited SIRT3 expression in both mRNA and protein levels, which upregulated acetylation levels of MTHFD2. Finally, cisplatin-induced acetylation of MTHFD2 leads to the decreasing of enzyme activity and NADPH production, which break the redox homeostasis in mitochondria.

The use of high-dose cisplatin in cancer therapy is frequently limited owing to its severe side effects in normal tissues, such as kidney, nerve, inner ear, and red blood cells^24,41,42^. Interestingly, we found that the acetylated K88 in MTHFD2 is dramatically decreased in tumor samples comparing with adjacent normal tissues, implying that targeting the acetylated K88 in MTHFD2 might be a potential approach for colorectal cancer treatment.

Besides, cisplatin resistance has been observed in many patients. The possible mechanisms of cisplatin resistance include increased detoxification in the tissues, changed in cellular uptake of cisplatin and increased in DNA repair^43,44^. Therefore, combination therapy of cisplatin with other cancer drugs has been applied as novel therapeutic strategies for many human cancers. Here, we found that SIRT3 protein levels were significantly negatively associated with acetylated K88 MTHFD2 in our colorectal tumor tissues, which suggested that inhibitors of SIRT3 or MTHFD2 might be an attractive drug to combinate with cisplatin for colorectal cancer treatment.

## Materials and methods

### Plasmid construction

Full-length cDNA of MTHFD2 was amplified by PCR and cloned into indicated vectors including pcDNA3.1-B(-)-Flag, pcDNA3.1-C(-)-Myc, pET15b, and pBABE vector using standard protocols. SIRT3 was cloned into pcDNA3.1-C(-)-Myc, pcDNA3.1-HA, and pQCXIH vector using standard protocols. Point mutations of the indicated constructions for MTHFD2 were generated by site-directed mutagenesis (KOD Plus Mutagenesis Kit TOYOBO). All constructions were confirmed by DNA sequencing before further applications.

### Chemicals

Nicotinamide was purchased from Sigma-Aldrich (Sigma 72345). Cisplatin was purchased from APExBIO(A8321).

### Cell culture and culture conditions

HEK293T, HCT116 (p53+/+), and Hela cells were obtained from the American Type Culture Collection (ATCC). HeLa, HEK293T, and HCT116 cells were cultured in Dulbecco’s Modified Eagle Medium (DMEM)/high glucose medium (HyClone) supplemented with 10% fetal bovine serum (FBS, BI), 100 units ml/1 penicillin and 100 µg ml/1 streptomycin (Sangon Biotech). Cultures were maintained at 37 °C in a humidified atmosphere containing 5% CO_2_.

### Cell treatment and transfection

Cell transfection was performed using polyethylenimine (PEI Polysciences) based on a 3:1 ratio of PEI (μg): total DNA (μg), according to the manufacturer’s protocol. Cell transfection for siRNA was carried out by Lipofectamine RNAi-MAX according to the manufacturer’s protocol. Synthetic siRNA oligo nucleotides were obtained commercially from Shanghai Genepharma Co, Ltd. Cells were grown to 50–70% confluency before treatments. For Nicotinamide (Sigma-Aldrich) and Cisplatin (Sigma-Aldrich) treatments, the reagents were diluted in DMEM (HyClone) serum-free and added into the cell media with different final concentrations and incubated for indicated time at 37 °C. The other treatments are described in figure legends.

And the sequences of siRNAs are:

siRNA_SIRT3#1: 5′-GCTTGATGGACCAGACAAA-3′;

siRNA_SIRT3#2: 5′-AAAGGTGGAAGAAGGTCCATATCTT-3′.

### Antibodies

The western blot primary antibodies against pan-Acetylated lysine (9441, 1:1000), SIRT3 (2627, 1:1000), Myc (2272, 1:1000) were purchased from Cell Signaling Technology. Antibody against Flag (SAB4301135, 1:5000) was purchased from Sigma-Aldrich. Antibody against Tubulin (AT819-1, 1:5000) were purchased from Beyotime. Antibody against MTHFD2 (sc-390708, 1:5000) was purchased from Santa Cruz.

To generate a site-specific antibody to detect the acetylated K88 of MTHFD2 (α-acK88, 1:500), synthesized peptide PASHSYVLNK (Ac) TRA (Shanghai HuiOu Biotechnology Co.Ltd) was coupled to KLH as antigen to immunize rabbit. Anti-serum was collected after four doses of immunization.

### Immunoprecipitation and western blot

For cell-based experiments, cells were washed twice in phosphate-buffered saline (PBS), scraped into PBS, pelleted, and resuspended in ice-cold 1% Nonidet P40 buffer for 30 min, containing 50 mm Tris-HCl (pH 7.5), 150 mm NaCl, 1% Nonidet P40, 1 mg/mL aprotinin, 1 mg/mL leupeptin, 1 mg/mL pepstatin, 1 mm Na_3_VO_4_, and 1 mm phenylmethylsulfonylfluoride (PMSF) (for testing acetylation level, needs contain 25 mm nicotinamide and TSA). Cell lysate was centrifuged at 12,000 × *g* for 15 min at 4 °C and then the insoluble fraction was discarded. The supernatant was incubated with anti-flag M2 agarose (Sigma A2220) for 3 h at 4 °C or with indicated antibody for 2 h followed by incubation with Protein-A beads (Santa Cruz) for another 2 h at 4 °C. After washing three times with ice-cold NP-40 lysis buffer and centrifuged at 2000 rpm for 2 min between each wash, the proteins were boiled by 1 × SDS loading buffer containing dithiothreitol. Then beads were centrifuged at 4 °C. The lysates were loaded on sodium dodecyl sulfate polyacrylamide gel and transferred to nitrocellulose membrane (GE Healthcare 10600002) for western blot analysis. The membranes were blocked for 1 h with tris-buffered saline with 0.1% Tween 20 containing 5% nonfat dry milk. For acetylation western blotting, 5% bovine serum albumin (BI) was used for blocking, and 50 mm Tris (pH 7.4) with 2.5% BSA was used to prepare primary and secondary antibodies. After incubated with primary antibodies, the membranes were washed and incubated with HRP-conjugated anti-mouse or anti-rabbit secondary antibodies and followed by detection using ECL Western blotting substrate (Bio-Rad).

### Generation of knockout cells using CRISPR/Cas9 genome editing

The sgRNA sequences targeting MTHFD2 were designed by CRISPR designer at http://crispr.mit.edu/. Gene deletion was verified by both DNA sequencing of genomic DNA and western blotting for cell lysate. The guide sequences targeting the human MTHFD2 are shown below:

MTHFD2#1: 5ʹ-CGCCAACCAGGATCACACTC-3ʹ;

MTHFD2#2: 5ʹ-GCCACACCTGAGTGTGATCC-3ʹ.

### Quantitative real-time PCR

The total RNAs were extracted from cells by using Trizol reagents (Ambion), and then the extracted RNAs were reverse-transcribed into cDNA by using the qPCR RT Master Mix with gDNA Remover kit (TOYOBO). The products were then used as templates for real-time PCR using the SYBR Green real-time PCR master mixes (TOYOBO). The real-time PCR were performed using CFX Connect Real-Time System (Bio-Rad). The quantity mRNA was calculated using the method ∆∆Ct, and the relative gene expression levels were normalized to ACTIN as an internal control. The real-time PCR primers used for different target genes are as listed below:

SIRT3 (forward): 5ʹ-CAGTCTGCCAAAGACCCTTC-3ʹ;

SIRT3 (reverse): 5ʹ-AACACAATGTCGGGCTTCAC-3ʹ;

ACTIN (forward): 5ʹ-TCCATCATGAAGTGTGACG-3ʹ;

ACTIN (reverse): 5ʹ-TACTCCTGCTTGCTGATCCAC-3ʹ.

### Recombinant human MTHFD2 expression and purification

The cDNA encoding human mitochondrial MTHFD2 (isoform 2; NCBI Reference Sequence: XP_006711987.1) was cloned into pET15b vector with a His-tag in N-terminal and transformed into *E. coli* BL21 (DE3). The transformed cells were grown in LB-medium containing 100 μg/mL ampicillin at 37 °C overnight. The overnight cultures were inoculated as 1/100 into 1 L of LB-medium containing Ampicillin (100 μg/ml). Cultures were grown at 37 °C OD600 of 0.6–0.8, protein expression was induced by addition of 0.2 mmol/L isopropyl β-d-1-thiogalactopyranoside. Cells were grown at 22 °C overnight, then the bacteria were harvested by centrifugation and washed in PBS. Cells were first dissolved in 30 ml binding buffer (20 mm Tris-HCl pH 8.0, 500 mm NaCl, and 30 mm imidazole), PMSF (0.5 mm) was added just before lysis. Then bacteria were lysed through high pressure disruption by a low temperature Ultra-High-Pressure Continuous Flow Cell Disrupter (JN-3000 plus), the cell lysate was centrifuged at 18,000 rpm for 1 h at 4 °C. The supernatant was loaded onto a 1 ml Ni-Sepharose column His Trap-HP (GE Healthcare) by a peristaltic pump and gradient eluted by an ÄKTA FPLC system (GE Healthcare) with elution buffer (20 mm Tris-HCl pH 8.0, 500 mm NaCl, and 500 mm imidazole). Fractions with eluted MTHFD2 were determined by western blot.

### In vivo site-specific incorporation of acetyllysine into MTHFD2 proteins

To generate a homogeneously K88-acetylated MTHFD2 construct for analysis, we used a three-plasmid (pET15b, pCDFpylT-1, and pAcKRS) system as previously described. We cloned wildtype mitochondrial MTHFD2 into pET15b and incorporated an amber codon at lysine 88 (AAG to TAG by site-directed mutagenesis with a KOD-Plus-Mutagenesis kit (TOYOBO)). The three plasmids were transformed into *E. coli* BL21, the overnight cultures were overexpressed in LB with ampicillin (100 mg/ml), kanamycin (50 mg/ml), and spectinomycin (50 mg/ml) induced with 0.2 mm IPTG in addition to 2 mm *N*-acetyl-lysine (Sigma A4021) and 20 mm nicotinamide to inhibit the activity of *E. coli* deacetylases. Cells were grown overnight at 22 °C. Cell culture, expression, and purification were performed as described above.

### In vitro acetylation assay

In vitro MTHFD2 acetylation assays were performed using His-tag MTHFD2 proteins purified from *E. coli* BL21. Reactions contain acetylation buffer (20 mmol/L pH 8.0 4-(2-hydroxyethyl)-1-piperazineethanesulfonic acid, 1 mmol/L dithiothreitol, 1 mmol/L phenylmethyl sulfonyl fluoride, and 0.1 mg/mL BSA), purified His-tag MTHFD2 proteins, and different concentrations of acetyl-coenzyme A (CoA) (A2056, Sigma-Aldrich). Reaction mixture was incubated at 30 °C for 15 min. Then the reaction was stopped by adding loading buffer, boiled and subjected to SDS-PAGE. Proteins were analyzed by Western blot.

### In vitro deacetylation assay

For the deacetylation experiments, recombinant K88-acetylated MTHFD2 purified from *E. coli* BL21 were incubated with purified SIRT3 or H248Y mutant in the presence or absence of 1 mmol/L NAD^+^ with deacetylation buffer (50 mm Tris-HCl pH 9.0, 4 mm MgCl2, 50 mm NaCl, 0.5 mm DTT, 0.5 μm TSA). Reactions were incubated at 30 °C for 2.5 h and then placed on ice for 15 min. For NAM treatment as control, reactions were pretreated with 10 mm nicotinamide for 10 min at 30 °C. Then the reaction was stopped by adding loading buffer and analyzed by western blot.

### MTHFD2 enzyme activity assay

Enzyme assays were performed after Yang and Mackenzie^45^ and Pawelek and MacKenzie^46^. Standard conditions buffer contained 25 mm MOPS (pH 7.3), 5 mm potassium phosphate (pH 7.3), 5 mm magnesium chloride, 2.5 mm formaldehyde, 0.2 mm tetrahydrofolate, 36 mm β-mercaptoethanol, and 0.6 mm NAD. Standard activity assays are reported as the average of three separate determinations performed in triplicate using a single fixed time point. Data of enzyme activities were from at least triplicate wells. The absorbance was measured by an EPOCH2 microplate reader (BioTek).

### NADPH levels assay

HCT116 cells were treated in the presence or absence of cisplatin for 24 h before experiments. Intracellular NADPH were determined in cells by using NAD(P)H Assay Kit (S0179, Beyotime). Cells (1 × 10^6^) were harvested and lysed in extraction buffer by freeze/thawing and lysed and centrifuged at 1,2000 rpm for 15 min. NADPH levels was measured by following the manufacturer’s instructions of Kit. Luminescence was detected using the BioTek Synergy microplate reader.

### GSH levels assay

Cells were treated in the presence or absence of cisplatin for 24 h before experiments. Cells were harvested and GSH levels was measured by following the manufacturer’s instructions of GSH Assay Kit (S0053, Beyotime).

### Cellular ROS assay

Cells were treated in the presence or absence of cisplatin for 24 h before experiments. Intracellular ROS production was determined in HCT116 cells by using 2’,7’-dichlorofluorescein diacetate (H2DCF-DA, Sigma-Aldrich) according to the manufacturer’s instructions. Cells were washed with PBS and incubated with 10 µm H2DCF-DA at 37 °C for 30 min to load the fluorescent dye. Cells were washed with PBS twice to measure the fluorescence (Ex.488 nm, Em.525 nm) by fluorescence microscope.

### Statistical analysis

Results were analyzed and graphed using Prism 7.0 software (Graphpad Software) or Microsoft Excel 2017 statistical program as indicated in the figure legends. Statistical analyses between groups were performed by unpaired two-tailed Student’s *t* test. *P < 0.05; **P < 0.01; ***P < 0.001; ****P < 0.0001; n.s., not significant for the indicated comparison.

## Supplementary information

Supplymentary Figure

Supplementary Figure Legends
