# Supplementary figures and images for: Cisplatin inhibits SIRT3-deacetylation MTHFD2 to disturb cellular redox balance in colorectal cancer cell
Source: Cell Death Dis. 2020 Aug 6;11(8):649. doi: 10.1038/s41419-020-02825-y (PMC7434776; doi:10.1038/s41419-020-02825-y)

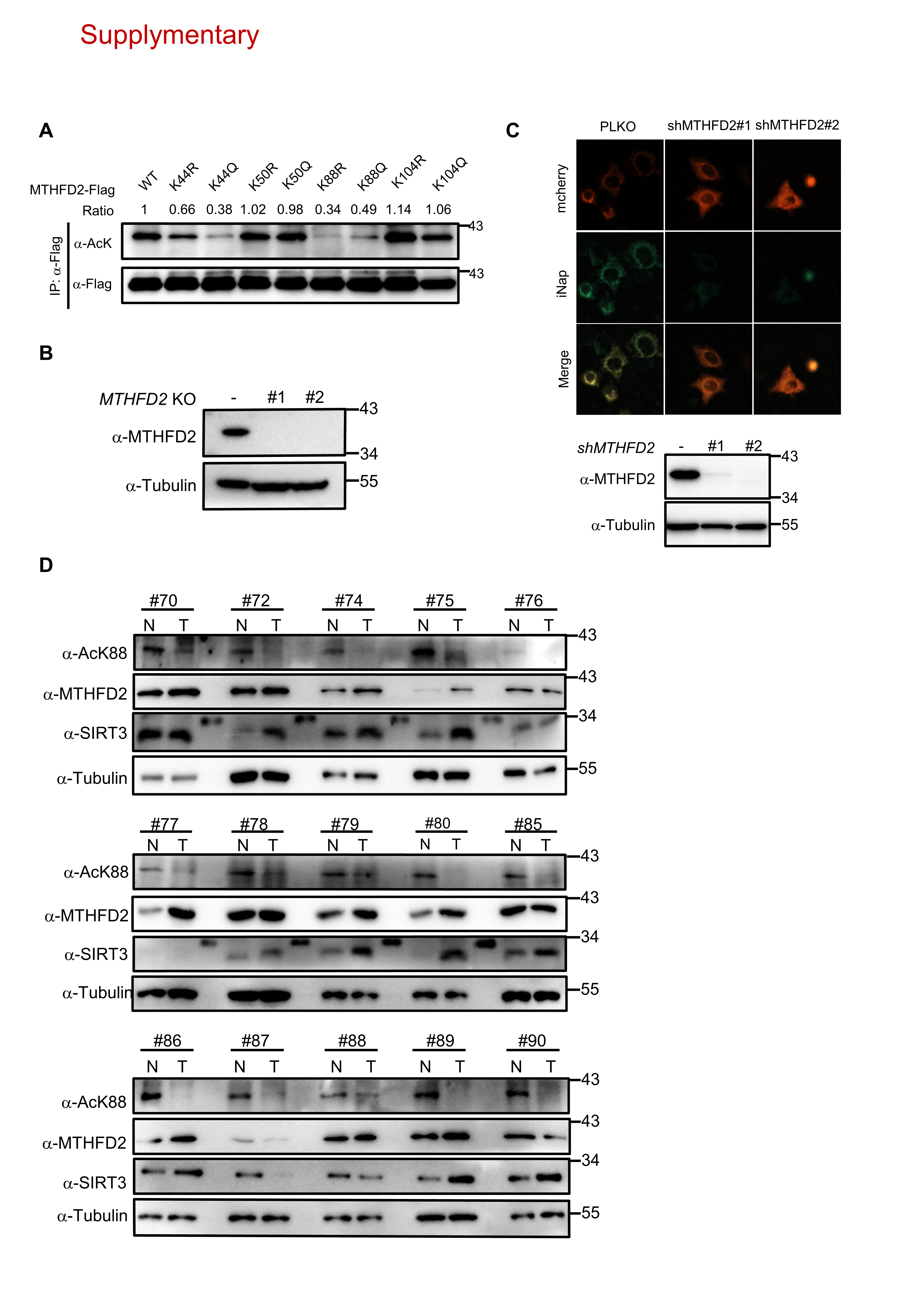

Supplement: Supplementary file 1 — Supplymentary Figure [file 41419_2020_2825_MOESM1_ESM.tif]
