# Supplementary material for: Cisplatin inhibits SIRT3-deacetylation MTHFD2 to disturb cellular redox balance in colorectal cancer cell
Source: Cell Death Dis. 2020 Aug 6;11(8):649. doi: 10.1038/s41419-020-02825-y (PMC7434776; doi:10.1038/s41419-020-02825-y)
Supplement: Supplementary file 2 — Supplementary Figure Legends [file 41419_2020_2825_MOESM2_ESM.docx]

**Supplementary Figure Legends**

1. K44, K50, K104 are not the primary acetylation site of MTHFD2. WT and K44R/Q, K50R/Q, K88R/Q, K104R/Q MTHFD2-Flag tag mutants were ectopically expressed into 293T cells, and the proteins were immunoprecipitated before being subjected to western blot for acetylation analysis.
2. Identification of HCT116 MTHFD2 KO cell lines. MTHFD2 was knocked out in HCT116 cells using CRISPR-Cas9 system.
3. Knocking down MTHFD2 decreases cellular NADPH level. iNap-NADPH sensors were expressed in shMTHFD2 Hela cells, and the NADPH levels can be detected by fluorescence 488nm.
4. In total, 15 pairs of tumor tissues (T) and adjacent normal tissues (N) were lysed. Protein levels of MTHFD2-K88Ac, MTHFD2 and SIRT3 were determined by direct western blot. Relative protein levels were normalized by α-Tubulin.
